# The Plea of Insanity

**Published:** 1898-08-13

**Authors:** 


### The Plea of Insanity.

At the meeting of the British Medical Association at Edinburgh a very interesting discussion took place in regard to the plea of insanity in criminal cases. One need hardly say that this a subject on which it would be possible to argue for many days without coming to any definite conclusion. But, in fact, the time has not yet arrived when any final conclusion is desirable. We are as yet too far from fulness of knowledge to make us wish to lay down rules, or to relieve that old institution, the British jury, of its ancient duty of deciding each case upon its merits, and upon the evidence laid before it. Nevertheless, in the discussion to which we have referred, certain points arose which will well bear consideration, showing as they do the directions in which opinions drift among specialists in mental disease. So widely is the notion current that the plea of insanity ought to relieve a man of the consequences of his acts that Dr. Mercier did well to insist upon the fact that, in the ordinary every-day work of dealing with lunatics in asylums, discipline is maintained by a system which cannot be properly described in any other terms than as a system of punishments. He said that for very many of their wrong acts the majority of lunatics ought to receive some punishment, and furthermore, he affirmed that punishment of the insane in some form or other was in practice in every institution for lunatics. If a patient on parole came in drunk would they not refuse him his parole next time, and time after time when he applied for it? When a woman had been fighting and smashing, would they not forbid her to attend the weekly dance? If a man were discovered pilfering or bullying, would they not stop his tobacco? Let them, he said, clear their minds of cant in this matter. They relied upon the temporary withdrawal of privilege to act as a check in preventing abuse in the future; "that was to say, they withdrew it as a punishment."

The practical application in the Courts of this general principle, as accepted in the asylums, would appear to be plain enough, namely, that while the punishment of an insane person should not be the same as that meted out to a sane one, and while in some cases an insane person might be held absolutely irresponsible for his actions, the mere fact that a patient was insane should not by any means be held to relieve him from the consequences of his acts. In other words, insanity, if proved, should be taken in mitigation of punishment rather than as showing that no punishment should be given.

Sir William Gairdner, when discussing the subject, remarked that at present the jury could say that a prisoner was guilty, or that through insanity he was not guilty. He desired that there should be a third alternative—guilty, but insane. The same view was put very forcibly by Dr. Blandford, who, speaking of the insanity which arises through drink, said that instead of hanging a man who committed a murder through drink he would send him, not to Broadmoor to smoke his pipe for the rest of his life, but to penal servitude. There can be no doubt that Dr. Blandford in saying this indicated a serious blot in the administration of justice. The difference between the two alternatives is too great, and it is fair neither to the judge, nor the jury, nor indeed to the prisoner himself, that no middle course should be available between sending a man to hang by the neck until he be dead, or sending him to "smoke his pipe at Broadmoor for the rest of his life."

As showing how varied are the views taken by different men on this subject of responsibility, we would refer for a moment to the remarks made by Dr. Sutherland, a Deputy Commissioner in Lunacy, in regard to crimes committed under the influence of drink. He did not think that in practice any injustice was done in the Courts to those who were insane, but he did think—nay, he knew—that gross injustice had been done to others who, through intoxication, were insane at the time the crime was committed. Intoxication was insanity of the purest kind, and yet the law did not recognise that intoxication *per se* was insanity at all. The consequence was that several men who had committed crimes under the influence of drink had had to pay the extreme penalty of the law. He further said that while drinking began voluntarily its voluntary character passed away, and it became involuntary, and he urged that the authors of intoxicated crimes ought not to suffer to the extent they did under the present state of the law, and that if any change in the law was to be made, it should take place not in regard to the deluded man, but in regard to the intoxicated man who committed crime of which he had no consciousness. We need hardly say how grave a matter such an extension as this of the doctrine of irresponsibility would be.

